# A Tale of Two Cities: Understanding Children's Cycling Behavior From the Socio-Ecological Perspective

**DOI:** 10.3389/fpubh.2022.864883

**Published:** 2022-03-29

**Authors:** Demet Yesiltepe, Rian Pepping, Fiona Chun Man Ling, Gavin Tempest, Steven Mauw, Mirka Janssen, Florentina Hettinga

**Affiliations:** ^1^Lancaster Institute for the Contemporary Arts, Lancaster University, Bailrigg, United Kingdom; ^2^Centre of Expertise Urban Vitality, Amsterdam University of Applied Science, Amsterdam, Netherlands; ^3^Department of Sport, Exercise and Rehabilitation, Northumbria University, Newcastle upon Tyne, United Kingdom

**Keywords:** health, built environment, physical activity, youth, cycling, social ecological model, active lifestyle, urban environments

## Abstract

The childhood obesity epidemic has persisted for over three decades, which has presented serious social, economic and health consequences worldwide. For researchers and policy makers alike, cycling has been a promising focus over recent years for developing long-term physically active lifestyles in urban environments, in addition to contributing to the global quest to combat climate change. Promoting cycling thus presents a win-win situation not just for individuals' well-being, but for multiple involved sectors such as public health, transport ministry and environmental agencies. For children, cycling promotes exercise engagement, active transport opportunities, motor skill development and social interaction. However, across European cities, there are considerable discrepancies in the uptake of cycling amongst children. To understand and subsequently promote children's cycling behavior, it is crucial that the complex social, physical and policy environment, and their interrelationships, are considered. Therefore, in this perspective article, we adopt the socio-ecological model to gain insight into how children's cycling behavior is shaped at the interpersonal, organizational and community level embedded within city policies, relevant to increase future cycling participation in children. Our perspective is based on a review of cycling policies of two European cities, Amsterdam (Netherlands) and Newcastle (UK), where stark contrasts in children's cycling participation can be observed. Our findings show that cycling policies in Amsterdam have mainly contributed to comprehensive organizational level changes, for example, cycling infrastructure development within the city, whereby these initiatives have made significant progress at the community level where cycling has become part of the “Dutch culture”. Hence, cycling is a more common transportation mode among children in Amsterdam than in Newcastle. In Newcastle, policies primarily focus on organizational or community level changes, and progress has recently been accelerated in response to COVID-19. In addition to differences, we have also identified similar challenges in the two cities, such as the urgency to support uptake of cycling for children with low socio-economic background or challenges related to cultural differences. We also propose a “shared (cycle-)path” for policy makers and researchers as working together is crucial in producing multi-component interventions at a policy level that recognize individual, as well as interpersonal, community and organizational factors.

## Introduction

The global rise in childhood obesity is partly due to the alarmingly low rates of children's physical activity (PA) participation. In 2012, the Lancet, one of the most respected medical and health journals, stated that physical inactivity has become a pandemic (1). In Europe alone only one in four 11-year-olds and only one in seven 15-year-olds took part in at least 1 h moderate-to-vigorous exercise daily in 2018 (2). In all countries, girls were less physically active than boys at both ages. With more than 80% of Europeans projected to reside in cities by 2030, the WHO has a strong agenda to promote physical activity in urban environments (3). Due to COVID-19, physical inactivity increased even more, and children's (especially children aged between 4 and 6) motor skill development was affected (4). Hence, increasing children's physical activity has become more urgent than ever. Increasing physical activity in children requires a wide range of interventions and strategies incorporating knowledge and experience at several different levels and settings. One aspect to explore for instance is active transport, which can provide children 15 min of extra movement per day (5). Therefore, cycling can be one of the pieces of the large physical inactivity puzzle. In recent years, cycling has gained popularity as a recreational activity and sustainable “greener” means of active transport. To increase cycling opportunities at first instance, an appropriate infra-structure is needed. Actions to increase cycling opportunities thus primarily occurred at a policy level within urban environments, e.g., providing infrastructure such as cycle ways or shared bicycle schemes. At the same time, at the individual behavioral level, the mental and physical benefits (6) of cycling (particularly improved motor skill development in children) (7) have become more evident. From the health and behavior change perspective, the alignment of policy and individual level changes must occur in tandem for the successful development and roll-out of public health initiatives to promote PA through cycling in children. The share of total trips that are completed by bicycle is high in Europe (for example it is around 9% in Germany or 19% in Denmark) compared to other westernized countries including Australia or North America (all 1%) (8). Cities have their own cultures, needs and geographical or urban characteristics to create safe and integrated cycling systems (9). These characteristics shape the cycling culture and thus, if we want to increase cycling participation, it is important to understand the current situation in different urban areas and the decisions that have been adopted by governments or local authorities. In this perspective, we will focus on two cities, Newcastle and Amsterdam, in Europe where the cycling rates are higher than many other continents. Amongst the European countries, the Netherlands has the highest cycling percentages (27%) while the percentage is much lower (2%) in the UK (10). This perspective article is oriented at understanding policies. Hence, we first describe current cycling policies in Newcastle and Amsterdam incorporating physical (e.g., infrastructure) and social (e.g., cycle training) factors relevant to promoting cycling in children and secondly apply the socio-ecological model to compare progress of cycling initiatives, in the two different cities. Policy interventions have been placed the context of a theoretical framework, the socio-ecological model, to be able to analyze and make recommendations. We highlight shared challenges of cycling initiatives in urban environments, that can be applicable to other cities, and provide recommendations for the development of effective, sustainable, and long-lasting public health interventions to promote PA through cycling in children.

## Urban Environments with Low Cycling Participation: Newcastle (UK)

In England, less than half of children walk or cycle to school despite nearly half of the population (42% of people) aged above 5 years owning or having access to a bicycle (10). In fact, bicycle ownership is much more likely among children aged 5–10 years old than other age groups (10). In 2019, 41% of all children (aged 5–16 years) usually walked to school, however, only 3% of all children cycled to school (11). Road safety concerns and traffic are common reasons for people not cycling more (11).

For Newcastle, there is a lack of city-wide cycling user data available. However, in a large-scale survey of primary school children (*n* = 4,775), 81% of students reported enjoying physical activities, yet when traveling to school, only 3% of students cycled compared to 54% of students taking passive transport (i.e., traveled by car, bus, taxi/minibus or public transport) (12). Considering the surprisingly low level of PA participation outside of school (only 9% of all children), the opportunity to cycle to school (and therefore increase daily PA) was not popular for reasons not reported. However, a previous dataset indicated that despite bicycle ownership between boys and girls being similar in participating Newcastle Schools (87 and 85%, respectively), the percentage of boys cycling to school was around 30% compared to around 25% for girls (13). Within the city of Newcastle, there are diverse socio-demographic characteristics (much like other urban environments), however, there are relatively little data available on specific factors (e.g., cultural and socio-economic status) which may impact cycling engagement and opportunities for different groups of children.

## Urban Environments with High Cycling Participation: Amsterdam (NL)

The Netherlands is well-known as a cycling country. According to the latest estimate, each person in the Netherlands owns more than 1.3 bicycles, which is unique in the world (14). In 2018, more than 25% of all movements in the Netherlands were completed using bicycles (15). The Netherlands has the highest percentage of movements using bicycles relative to the world population (16).

In the Netherlands, children cycle more than adults in terms of percentage of trips they complete by bike. Almost half of all movements (48%) by children aged under 18 years are cycling related (17). Of all age groups, young people aged 12–18 years use their bicycles the most. With an average of 6.2 kilometers per day, youngsters also cover the longest distance (18). However, in recent years, the number of cycling children has declined, partly because only half of children aged 4–12 years have learnt to cycle in urban areas (19). In Amsterdam, more than 16,000 primary school children cannot cycle (20). Most of these children are from families with a low economic status. Furthermore, among families with a low economic status, bicycle ownership and cycling skills are more limited which further hinders cycling opportunities (20).

## Applying the Socio-Ecological Model to Compare Cycling Initiatives in Urban Environments

**Figure 1:**
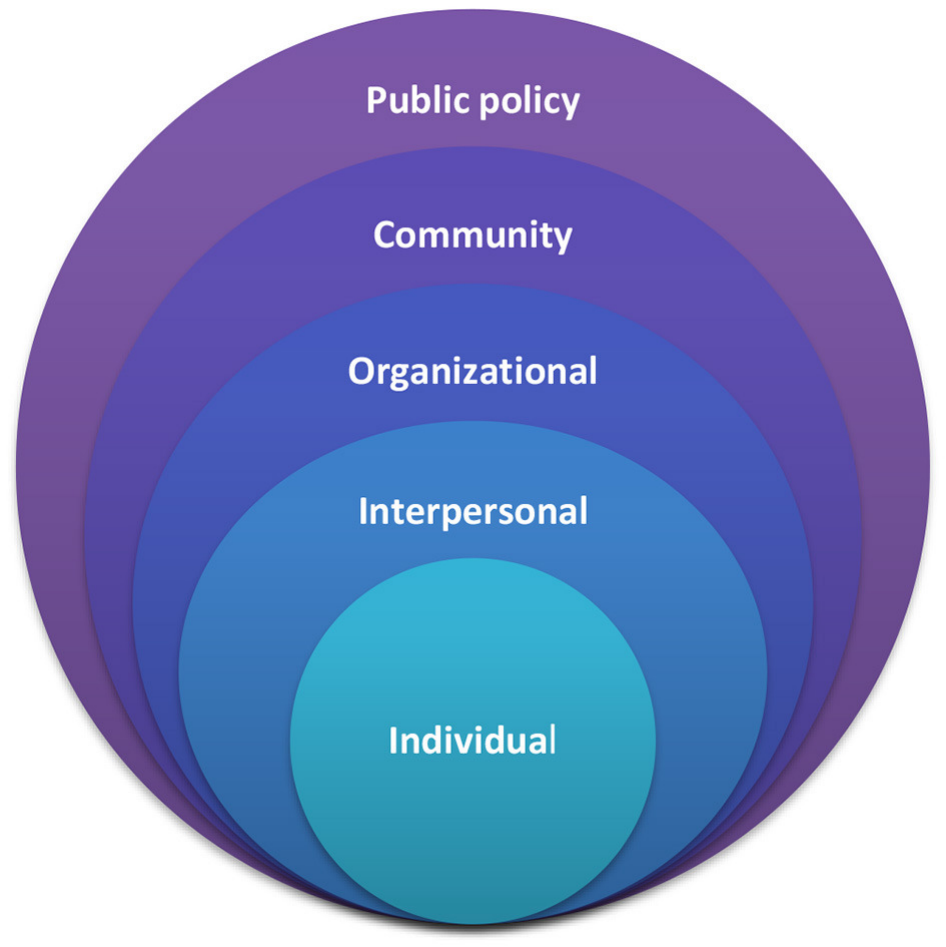
The socio-ecological model (24).

In this perspective article, we compare the impact of cycling policies in Newcastle and Amsterdam on children's cycling behaviors using the socio-ecological model (21, 22). The socio-ecological model (SEM) considers how the broad political and environmental factors shape individual and interpersonal characteristics (23). The model is comprised of individual (e.g., knowledge and attitude), interpersonal (e.g., immediate physical environment and social unit), organizational (larger environments and (in)formal organizations), and community (e.g., demographic, ethnic, religious characteristics) level changes that are embedded within the policy environment (Figure 1) (24). Various preceding studies used the socio-ecological model in physical activity studies as it helps health professionals plan, implement, and/or evaluate physical activity interventions (25). In addition, it helps to focus not only on individual characteristics but also the social and physical environment context that can include family, friends, or formal and informal organizations and facilities that promote or prevent physical activity (26) and allows to provide a holistic understanding of how policy interventions interact with a range of personal/social factors, which is needed to be able to understand what are appropriate interventions at what time, in which city and targeting who. Increasing cycling behaviors among children is complex since many factors, including personal factors such as cycling skills and environmental factors such as infrastructure, affect it. Hence, we use the socio-ecological model to capture the factors influencing cycling behavior in children at five levels: individual, interpersonal, organizational, community and policy. Within the context of cycling in children, policy makers and researchers usually focus on physical environment and individual level factors. The socio-ecological model suggests that the combination of individual, social, and physical environmental factors explain PA participation best and hence, the SEM is particularly appropriate for studying PA (27). This model allows us to explore policy interventions at different levels, and provide insights and understanding into the differences in children's cycling participation in different cities, as well as in promotion policies targeting an increase in children's cycling participation. These insights can inform future intervention strategies. Hence, during analyzing the reports and decisions for Amsterdam and Newcastle, we aimed to understand the levels targeted in SEM.

### Newcastle: Getting Wheels on the Road

Policies in Newcastle have mostly focused on getting people (not just children specifically) to cycle more. At the organizational level, policies aimed to create continuous, safe, direct routes where cyclists can travel separated from the vehicles (28), and “school streets”: closing streets to through traffic and having parking restrictions at school pick-up and drop-off times (29), or closing bridges to traffic in order to create safer routes for children (30, 31). Community level activities were supported, such as, “walk to school week” (32), whereby parents and children were invited to actively commute to school. In addition, Newcastle Healthy School program promoted health and well-being of children and staff, whereby schools provided evidence of their health promoting practice or undertook a 2-year program of activity on a public health issue (26).

Interpersonal or individual level changes have also been targeted, such as through the “Active Newcastle” program to promote physical activity and reduce health inequalities for different age or gender groups (33), nationwide cycling festivals (34), a “Fix your bike” voucher scheme launched by the Department of Transport (35) as well as free cycling trainings for schools offered by the Newcastle city council (36). However, few policies targeting individual and interpersonal level changes in Newcastle have been implemented.

### Amsterdam: Keeping the Wheels Turning

In Amsterdam, organizational level changes are ongoing and are far more advanced than in Newcastle. While safe cycling routes are already present throughout the city, further improvements to current infrastructure to better connect different parts of the city have been proposed, so that new routes will be added and existing routes will be modified (37). At the community level, a main goal is to strengthen the cycling culture amongst the low socio-economic groups and non-western background people (38) due to their low cycling uptake. In addition, to promote cycling, there are specific schemes at schools. For example, parents can declare costs for cycling or parents from lower income groups can buy bikes with discounts (28).

At the individual and interpersonal level changes, the aim is to increase children's and their parents' cycling skills so that the parents can be role models to encourage cycling with their children. All equipment such as bicycles and helmets are provided by the executive party “Verkeersplein Amsterdam” and the cycling activities target children who cannot learn cycling from home (39).

## Shared Challenges: Riding Towards Promoting Children's PA Through Cycling in Urban Environments

**Figure 2:**
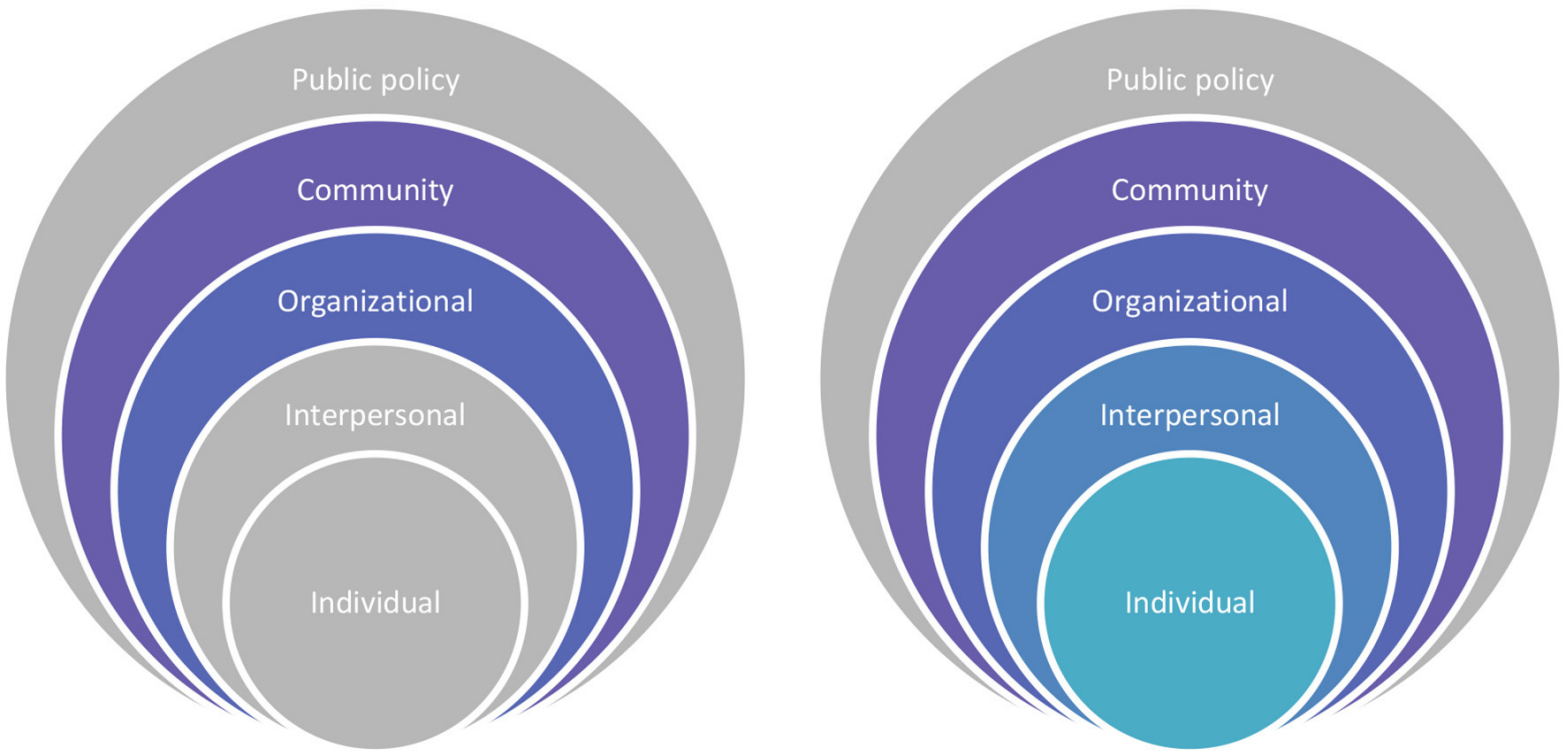
Socio-ecological model and the levels covered by the policies (on the left, policies in Newcastle and on the right, policies in Amsterdam). Colored parts of the figure show the targeted levels and gray parts show the levels that are not (fully) targeted.

The policies in each city target different levels of the social ecological model (see Figure 2). Amsterdam city already has a high number of cyclists (children included), and the infrastructure is already well-developed to support cycling. Hence, policies mostly focus on individual and interpersonal level changes. On the other hand, in Newcastle, cycling infrastructure is yet to be developed in order to support cycling behavior. Hence, policies focus on organizational and community level changes yet interpersonal and individual level changes are warranted. In addition to generating a sound cycling infrastructure, policies that are based on knowledge of the factors that impact cycling behavior in children (with or without disabilities) from low socio-economic groups, ethnic minorities and different gender groups are critical. Despite that Amsterdam has already got the wheels turning, more concerted strategies from the government in both cities are called for to help improve the cycling culture amongst their children to promote PA through cycling. To target children specifically, both cities appear to have (to some extent) collaborations with schools to promote cycling initiatives acknowledging the need to implement individual and/or community level changes.

Additionally, policies should target the most influential and the most modifiable factors that impact cycling behavior, such as bike parking, schemes to promote cycling and parental perception of safety. It is crucial to identify such factors that are meaningful to policy makers. Until recently, cycling and walking have not been priorities of transport plans for some time in the two cities. This has recently changed, especially in Amsterdam (38); however, there is still a lot that can be done. Rather than having plans focused on reducing vehicles on the road [to become more “green” and reduce CO_2_ emissions (40, 41)], the focus of plans should be shifted toward making cycling (and walking) more appealing and encouraging PA, particularly for children and young people. Recent attempts show that it is possible to create large car-free areas to create safe and healthier environments for pedestrians and cyclists (42). This shift from car-dominated cities to car-free areas/regions can be adapted to any country/city in the world (organizational level change), which can help improve interpersonal and individual level changes.

## Discussion: A Shared “Cycle-Path” for Policy Makers and Researchers to Develop and Implement Effective Cycling Initiatives to Promote Life-Long Physical Activity in Children

Seldom do the “cycle-paths” of policy-makers, who implement public health initiatives, and researchers, who devise behavior change initiatives, co-create PA interventions and design implementation studies, run in parallel. It is a common practice for government to implement strategies before consultations are sought from the targeted population or from researchers, while an evidence-based approach, involving stakeholder's input, would likely increase effectiveness. At the moment, the policy regarding promoting cycling seems to be limited as it does not cover all determinants as proposed in SEM. Public policies mainly focus on external conditions relevant to cycling policy (e.g., infrastructure, parking spots or shared bikes). Though this is very important, particularly in the UK where cycling infra-structure is less developed than in the Netherlands, policies should also target the social factors that impact cycling behavior, self-efficacy or skills. As stated before, policies focus on keeping the wheels turning, rather than promoting cycling among citizens who are not yet able to, or unwilling to cycle. For example, there is not much effort to promote cycling among children from low socio-economic groups in both cities. Currently, there are cycling promotion events mostly at school level to learn more about cycling and schools can participate these events voluntarily. But this is not embedded in policies yet. This requires more cooperation between departments that work on cycling and other active travel modes (e.g., department of health, planning). Besides, more research is needed to identify the barriers or motivations for children who do not cycle. This can help us determine the effectiveness of current interventions.

In health research, evidence-based practice is a key term for decision-making in medicine (43). It involves considering 1. target population/patient perspectives, 2. expertise of professionals, and 3. available scientific evidence, to make adequate decisions about healthcare. For successful uptake of behavior change strategies (at the crux of successful policy implementation), it is imperative that policy-makers and other stakeholders, such as children, parents and schools, are involved from the outset (44) and available scientific evidence is used as input for interventions (43). Working together is crucial in producing and evaluating multi-component interventions at a policy level that recognize individual, as well as interpersonal, community and organizational needs that can lead to positive behavior change at a societal level, and, in this case, promote PA through cycling in children. For both Amsterdam and Newcastle, it is time to focus on both the external conditions as well as the social aspects to promote cycling.

## Data Availability Statement

The original contributions presented in the study are included in the article/supplementary material, further inquiries can be directed to the corresponding author/s.

## Author Contributions

DY and RP collected the data and completed the analysis. DY, RP, FL, GT, SM, MJ, and FH provided critical input to the manuscript. All authors contributed to the conceptualization of the study. All authors contributed to the article and approved the submitted version.

## Funding

This research was funded by pump priming fund that supports collaborative research projects between Northumbria University (NU) and Amsterdam University of Applied Sciences (AUAS).

## Conflict of Interest

The authors declare that the research was conducted in the absence of any commercial or financial relationships that could be construed as a potential conflict of interest.

## Publisher's Note

All claims expressed in this article are solely those of the authors and do not necessarily represent those of their affiliated organizations, or those of the publisher, the editors and the reviewers. Any product that may be evaluated in this article, or claim that may be made by its manufacturer, is not guaranteed or endorsed by the publisher.
